# Assessing the Quality of Narrative Feedback in Entrustable Professional Activities Using the EFeCT Feedback Scoring Tool

**DOI:** 10.1111/tct.70295

**Published:** 2025-12-08

**Authors:** Rebecca Lee, Neil Dhami, William Gibson, Deena M. Hamza, Anna E. Oswald, Mandy Moffat

**Affiliations:** ^1^ Department of Medicine University of Alberta Hospital Edmonton Alberta Canada; ^2^ Centre for Medical Education, School of Medicine University of Dundee Dundee UK

**Keywords:** entrustable professional feedback, EPAs, narrative feedback, undergraduate medical education

## Abstract

**Background:**

Competency‐based medical education (CBME) is the cornerstone of undergraduate training in Canada. Entrustable professional activities (EPAs) assess competency in professional tasks, with narrative feedback being a key component. There is currently a lack of published literature on the quality of narrative feedback in EPA observations in undergraduate medical education. This study explores the quality of narrative feedback in EPA observations provided to medical students.

**Methods:**

The quality of narrative feedback in a random sample of anonymised EPA observations from Year 3 students was evaluated using the Evaluation of Feedback Captured Tool (EFeCT). The EFeCT tool explores five facets of quality feedback, with a score of five indicating high‐quality feedback. Three evaluators independently assessed the quality of narrative feedback using the EFeCT tool. Any differences in score were resolved through discussion to reach consensus.

**Results:**

In the 2022–2023 academic year, 15,240 EPA observations were completed for year 3 students. A subset of 748 observations was analysed. Of these, one scored 0, seven scored 1, 33 scored 2, 115 scored 3, 151 scored 4 and 441 scored 5 on the EFeCT tool. The mean EFeCT score was 4.3.

**Conclusions:**

Overall, the majority of EPA narratives provided moderate to high‐quality feedback to students. However, variability was evident, and many EPA narratives were missing one or more elements of high‐quality feedback. This could result in significant implications for learner development. Addressing contextual factors such as clinical workload and creating faculty development opportunities may support faculty in providing high‐quality narrative feedback.

## 1 Introduction

Competency‐based medical education (CBME) is a key component of undergraduate medical training in Canada [1]. It fosters an environment where learners can achieve core competencies in a variety of tasks and skills that are required for working as a physician [2]. In 2012, the Future of Medical Education in Canada Postgraduate Implementation Project recommended the development of a clearer, more progressive transition between undergraduate medical training and postgraduate training. Recommendations included collaboration between undergraduate and postgraduate committees to identify competencies that residents would be expected to perform without direct supervision on the first day of residency [3]. Based on these recommendations, the Association of Faculties of Medicine of Canada created 12 core Entrustable Professional Activities (EPAs) in which all graduating medical students in Canada are expected to demonstrate competence [4]. These 12 core EPAs were incorporated into the MD programme at this institution in Canada in 2021 (Appendix S1). The EPA observation forms contain a rating scale and free text boxes in which faculty can add narrative feedback.

Narrative feedback provided in EPA observations helps to support the rating scale score given by faculty while also serving to provide formative feedback to students on both their strengths and on areas for improvement [5]. The narrative feedback provided to students aligns with Hattie and Timperley's feedback model, which emphasises the importance of feedback in enhancing learning [6]. Hattie and Timperley identified three feedback questions, which should be addressed in order to provide high‐quality feedback: ‘where am I going’, ‘how am I going’ and ‘where to next’ [6].

Student perceptions of quality feedback align with Hattie and Timperley's model. They report that they want narrative feedback to be individualised and specific. They would like specific feedback on what to improve and specific feedback on *how* to improve [7, 8]. Appropriate coaching strategies can help students move up the rating scales [9].

*They would like specific feedback on what to improve and specific feedback on* how *to improve*.

Faculty feel that the narrative comments in EPA observations are important for students' clinical development and that this feedback should be individualised to the student [10]. Although the rating scale language provides some context and information, faculty feel that the narrative component of the EPA observation is the most important component [10, 11]. This may be contributed to by the fact that faculty have to recall specific aspects of the clinical performance that they feel are important in order to provide narrative feedback. Rating scales on the other hand may be made more on ‘gut instinct’ [11].

Research evaluating the quality of narrative comments in EPA observations has primarily focused on postgraduate medical training and has demonstrated variability in the quality of narrative feedback [12, 13, 14]. The quality of narrative feedback in EPA observations may differ between undergraduate and postgraduate medical training for a number of reasons. In undergraduate medical training, learners have limited clinical experience and their tasks tend to be lower stakes [15]. As a result, feedback to undergraduate trainees may be more prescriptive and task oriented compared with postgraduate trainees where feedback may be more reflective and evaluative. EPA observations in postgraduate training tend to focus on more complex tasks such as managing a patient with multiple comorbidities and as a result their feedback may be more nuanced and comprehensive, addressing both clinical and nonclinical competencies such as leadership and teamwork [15].

Published literature focusing on the quality of narrative feedback in EPA observations in undergraduate medical education demonstrates differing results. One study examined the quality of narrative feedback in EPA observations in a psychiatry clerkship at one institution in Switzerland as part of a larger study looking at EPA observations. This study concluded that most EPA observations contained high‐quality narrative feedback [16].

*Published literature focusing on the quality of narrative feedback in EPA observations in undergraduate medical education demonstrates differing results*.

A more recent study at one institution in Australia concluded that the narrative comments in EPA observations in undergraduate medical education often provided feedback about what was done well but rarely provided feedback on areas for improvement [17]. This study explored the quality of narrative comments in a large number of EPA observations but only explored 2 of the 14 different EPAs in use at that institution [17].

Although there are examples of published literature exploring the quality of EPA narrative feedback in undergraduate medical education, there continues to be a paucity of literature dedicated to this topic and the published literature has drawn differing conclusions [16, 17]. The differing results may be due to variations in how narrative feedback quality is defined and measured. They could also be due to variations in feedback culture in different specialties. This study contributes to this topic by evaluating the quality of narrative feedback provided in EPA observations.

There are several tools that can be used to assess the quality of narrative feedback in workplace assessments. Completed Clinical Evaluation Reports are designed to assess narrative feedback at the end of a rotation [18]. However, this tool is cumbersome to use for shorter workplace‐based assessments such as EPAs and some of the questions are not applicable to EPAs [18, 19]. The Quality of Assessment of Learning Tool and the Quality Improvement Instrument were both designed to assess the quality of narrative feedback following shorter workplace assessments [19, 20]. However, these tools focus predominantly on ‘how am I going’, with less focus on ‘where to next’. They do not allocate specific points on ‘where am I going’ [19, 20]. The Evaluation of Feedback Captured Tool (EFeCT) is a tool designed to assess the quality of narrative feedback without requiring any specific feedback structure. It aligns well with Hattie and Timperley's feedback model [6, 19]. It has been demonstrated to be equally effective when used by clinical and non‐clinical raters [19]. For these reasons, the study team elected to use the EFeCT tool in this study.

## 2 Research Aim

This study aims to assess the quality of narrative feedback provided in undergraduate EPA observations using a published quality rating tool.

## 3 Materials and Methods

### 3.1 Participants

Narrative feedback from anonymised EPA observations from the 2022–2023 academic year for third‐year students at one institution in Canada was extracted from the undergraduate online student portfolio by a programme faculty member who was not affiliated with this study. Third‐year students at this institution rotate through core clerkships including internal medicine, surgery, paediatrics, obstetrics and gynaecology, family medicine and psychiatry. Fourth‐year students were excluded in order to ensure the study remained feasible within the study timeframe. The EPA observations were anonymised and identifying features such as student and faculty names were removed prior to distribution to the study team.

On review of the EPA observation numbers from year three for the 2022–2023 academic year, 164 students completed 15,240 EPA observations. As no previous studies have employed the EFeCT tool in an undergraduate medical student population, no data were available to guide a sample size calculation. A sample size of 748 (approximately 5%) was selected. This sample size resulted in a 95% confidence level, and a 3.49% margin of error [21]. This sample size was felt likely to provide a reasonable estimate of the distribution of the scores across EPA observations while still being feasible to achieve within the timeframe of this study.

### 3.2 Data Capture and Procedures

A total of 748 EPAs were randomly selected for evaluation. To ensure representativeness, we first calculated the proportion of completed observations for each EPA type, then selected the same proportion for analysis. Each observation was assigned a sequential number, and an online random number generator was used within each EPA type to select the evaluations. For example, EPA 9 accounted for 242 observations (1.6% of the total). Twelve of these were randomly selected for analysis using an online number generator.

All of the EPA observations had narrative feedback because the observation form is unable to be submitted until all sections of the form have been completed.

The EPA observation form includes the following prompts to help improve the quality of narrative feedback:

- ‘Describe case’;
- ‘Please describe what the student is doing well and why they should keep doing it’;
- ‘Please describe something the student can do to improve for next time’.

The quality of the narrative feedback in the selected EPA observations was evaluated using the EFeCT tool. This tool contains five criteria that are considered to be essential components of high‐quality feedback [6, 19]. Each of the criteria is assigned a numerical value—‘1’ if the criterion has been met or ‘0’ if the criterion has not been met. This results in a score of 0 to 5, with higher scores being representative of higher quality feedback. The EFeCT tool allocates points for the task being performed (‘where am I going’), how the learner did (‘how am I going’) and what was done well or needs improvement and how it was done well or can be improved (‘where to next’). It also allocates a point for context which is important to help the learner recall the encounter [19]. The EFeCT tool is shown in Table 1.

**TABLE 1:** EFeCT tool.

**Instructions:** This tool is for scoring the quality of written feedback captured on assessment forms. Each of the criteria below is identified as an essential element of high‐quality feedback.			
Scores for written feedback will be cumulative, resulting in a maximum score of 5. Criteria are not hierarchical; for example, it is possible to receive a score for Criterion F even if Criterion C has not been met. If comments are teaching only, with no reference to the performance of the learner, then the feedback ranks 0. Comments that only log an encounter (with no information at all about learner involvement) rank 0.			
**Criterion**	**Element**	**Written feedback**	**Score**
A		No written feedback provided at all	0
B	**What** did the learner do?	Some information about learner performance is provided, even if it is neutral (‘diagnosed patient’, ‘performed’). Using the assessment form etc. as a teaching tool is **not** feedback about learner performance	1
C	Context: **when**, **who**, **where** (any or all)	There is a cue about the type of patient (i.e., cardiac, mental health) and/or their demographics or context/symptoms (i.e., gender, age, ‘demanding patient’, ‘Type II diabetic’) to help the learner remember the encounter **OR** If feedback is about a procedure or general skill (charting, EMRs etc.), there is sufficient information about the procedure or general skill to help the learner remember the context of the feedback.	1
D	**How** did the learner do?	The feedback specifically mentions if the task was well done, needs to be worked on, or is a concern.	1
E	**What** was done well or needs improvement (task specificity)	There is feedback about a specific, tangible skill(s) to improve on or continue performing for future scenarios.	1
F	**How** was it done well or **how** can it be improved?	If something was positive, the feedback outlines which specific element of the visit was positive. The learner should be able to replicate the specific skill in the future. **OR** There is information to guide the learner on how to do better in future (fix an error or improve performance).	1

#### 3.2.1 EFeCT Tool [19]

All data were stored on this institution's encrypted, secure servers. Only the principal investigator and coinvestigators had access to the data. Ethical approval was obtained from the institutional Research Ethics Board and Trainee Research Access Committee.

### 3.3 Data Analyses

The quality of the narrative feedback for each EPA observation was scored on a numerical scale from 0 to 5 using the EFeCT tool. Three evaluators were involved with scoring the narrative feedback (R.L., W.G. and N.D.). Investigator triangulation with three evaluators reduces the impact of subjectivity and potential biases, resulting in improved reliability and validity of results [22]. To assess inter‐rater reliability, an initial sample of 22 EPAs was evaluated by all three evaluators independently. The intraclass correlation coefficient was then calculated, which showed an average measure of 0.67 with a lower bound confidence interval of 0.36 and an upper bound confidence interval of 0.84. These results indicated moderate inter‐rater reliability but did not meet the generally accepted cut off of greater than 0.70 for good inter‐rater reliability [23].

As a result of not meeting the criteria for good interrater reliability, the three evaluators met to review all of the narrative comments from these initial 22 EPAs. A consensus was made on how to score narrative comments using the EFeCT tool. A second sample of 24 EPA observations was then reviewed and scored by each of the three evaluators independently. The intraclass correlation coefficient was then recalculated. This demonstrated an average measure of 0.96 with a lower bound confidence interval of 0.93 and an upper bound confidence interval of 0.98. These results indicated excellent inter‐rater reliability [23].

Subsequent to this, the three evaluators assessed and scored each of the 748 EPAs using the EFeCT tool. Any scoring differences were resolved by discussion and consensus between the three evaluators.

Once all of the EPAs had been evaluated, the average intraclass correlation coefficient between the three evaluators was calculated. This demonstrated an average intraclass correlation coefficient of 0.66 with a lower bound 95% confidence interval of 0.58 and an upper bound 95% confidence interval of 0.73. This indicates a moderate intraclass correlation coefficient indicating moderate inter‐rater reliability [23].

## 4 Results

A total of 748 EPA observations were analysed. EPA 1 (history and physical) was the most common type of EPA observation completed, representing 25.4% of all completed EPAs. Other commonly completed EPA observations included EPAs 5 (assesses management plans), 6 (assesses oral/written reports) and 11 (assesses procedures), representing 11.9%, 18.3% and 13.9% of completed EPAs, respectively. EPA 10 (safety and improvement) was the least common EPA completed, representing only 0.4% of completed EPA observations. EPA 8 (urgent/emergent care) was also less frequently completed, representing 0.9% of completed EPA observations.

**FIGURE 1:**
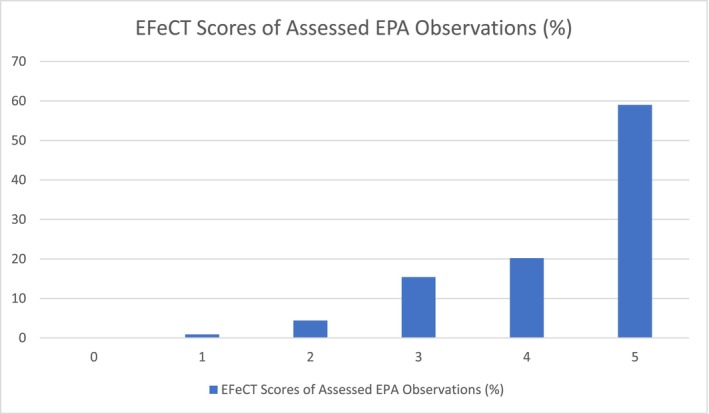
EFeCT scores of assessed EPA observations.

All of the EPAS were scored using the EFeCT tool. All EPA observations (100%) contained narrative comments. Figure 1 illustrates the EFeCT scores of the assessed EPA observations.

The mean score was 4.3, the median was 5 and the mode was 5.

Table 2 outlines the number of EPA observations that lost points on each specific domain on the EFeCT tool.

**TABLE 2:** Number of EPAs meeting EFeCT criteria.

EFeCT criteria	Number of EPA observations	Percentage of EPA observations assessed (%)
A—no written feedback provided	0	0
Lost point on B—what did the learner do?	33	4.4
Lost point on C—context	22	2.9
Lost point on D—how did the learner do?	39	5.2
Lost point on E—what was done well or needs improvement?	145	19.4
Lost point on F—how was it done well or how can it be improved?	273	36.5

The majority of EPA observations that lost points did so on ‘F’, which represents content about how the learner has completed an activity well or how it can be improved, with 273 (36.5%) of EPAs not meeting this criterion. This indicates that the feedback did not provide suggestions on how to improve performance or which specific skill the student should continue doing in the future. The second most common point lost was ‘E’, which represents content about what the learner did well or what needed improvement, with 145 (19.4%) of EPA observations not meeting this criterion. This indicates that there was no feedback on a specific skill to improve on or to continue performing for future scenarios provided in these EPAs. It should be noted that some of the EPAs lost points in multiple EFeCT tool criteria, whereas others lost points in only one criterion.

## 5 Discussion

This study contributes to the literature by quantifying the quality of the narrative feedback received in EPA observations across 1 year at one institution in Canada. Overall, narrative feedback quality was moderate to high, with 59% of assessed EPA observations meeting all EFeCT criteria for high‐quality feedback. This contrasts with a recent study that reported lower rates of improvement‐oriented feedback [17]. One reason for this difference in results may be that EPAs had been in use for a longer period of time at this institution compared with the recently published study [17]. This may have resulted in faculty feeling more comfortable in providing narrative feedback. Another possible explanation is that this institution requires the completion of three narrative text boxes (case description, strengths and areas for improvement) for EPA observation form submission, a structure that may enhance feedback quality. Third, this institution requires EPAs to be completed within 2 weeks, whereas institutions without expiry dates risk delayed documentation, reducing the specificity and actionability of feedback.

*Overall, narrative feedback quality was moderate to high, with 59% of assessed EPA observations meeting all EFeCT criteria for high‐quality feedback*.

Results from our study indicate that the three feedback questions in Hattie and Timperley's feedback framework were addressed the majority of the time. However, 41% of the assessed EPA observations omitted at least one element of high‐quality feedback, most commonly actionable feedback. This aligns with published literature which suggests that ‘where to next’ feedback is frequently omitted despite being most impactful [6, 17].

*However, 41% of the assessed EPA observations omitted at least one element of high‐quality feedback, most commonly actionable feedback*.

Variable quality of narrative feedback may reflect faculty workload, and competing demands, particularly for faculty supervising multiple learners [12, 14, 24]. In contrast, faculty who work one‐on‐one with students for a more prolonged period of time may provide more individualised and specific feedback [25, 26].

Faculty and trainee characteristics may also influence the quality of narrative feedback. Although this study did not evaluate the impact of faculty or student gender on the quality of the narrative feedback, previous research demonstrates that male faculty tend to deliver reinforcing feedback [14]. It has been reported that male students are more likely to receive strategies for improvement [17]. Junior faculty are more likely to provide specific feedback [14].

Faculty development can improve both specificity and quality of narrative feedback [27, 28, 29]. This underscores the importance of ongoing faculty development as a strategy to improve narrative feedback in EPA observations. The optimal format for faculty development is uncertain, but it has been suggested that longitudinal faculty development, which can incorporate repeated opportunities for practice, reflection and feedback, is often better at changing practice than single faculty development sessions [30]. Longitudinal faculty development also allows for reinforcement of key concepts and calibration across faculty.

Although the EFeCT tool was originally developed for programme‐level evaluation of narrative feedback quality in assessment forms, it also has potential applications in faculty development. Within faculty development sessions, the tool could provide a structured framework for analysing and delivering feedback, with faculty reviewing sample narratives to enhance feedback literacy. It could also support peer‐to‐peer learning by enabling faculty to evaluate each other's feedback. Additionally, integrating the tool into EPA electronic platforms could offer real‐time prompts and reinforce high‐quality feedback practices, potentially improving the overall quality of narrative feedback.

### 5.1 Limitations

This study has a number of limitations. This study was a single‐centre study; however, the study team feels that given the transparency of the methodology, the approach and learning are likely transferable to other institutions. This study did not differentiate between different clerkship disciplines such as internal medicine, paediatrics or surgery. This was deliberately not assessed in order to protect the anonymity of students and faculty. However, as a result, it is unclear if different clerkships provide differing qualities of narrative feedback in EPA observations.

This study only assessed narrative feedback in EPA observations and so was not able to assess any verbal or informal feedback that may have been received by students.

### 5.2 Implications

Despite the overall quality of narrative feedback in EPA observations being moderate to high quality, the frequent omission of actionable ‘where to next’ feedback reflects a persistent gap with potentially significant implications for learner development. Addressing contextual factors such as clinical workload, faculty time pressures and continuity with learners may support faculty in providing high‐quality feedback.

Future directions include leveraging faculty development to enhance the specificity and actionability of narrative feedback provided in EPA observations. Longitudinal approaches that allow for practice, reflection and calibration are more likely to produce sustained change than one‐off sessions. The EFeCT tool may serve as a valuable framework for such initiatives, with potential applications in structured workshops, peer‐to‐peer learning and integration into electronic EPA platforms to provide real‐time feedback prompts. Future research should explore how longitudinal faculty development, including the use of tools such as the EFeCT tool, influences the provision of high‐quality narrative feedback in EPA observations.

*Future directions include leveraging faculty development to enhance the specificity and actionability of narrative feedback provided in EPA observations*.

## 6 Conclusions

Overall, the quality of narrative feedback in EPA observations for undergraduate medical students at a single institution in Canada was moderate to high. However, there was significant variability and many EPA observations were missing one or more elements of high‐quality narrative feedback, indicating a need for improvement. Faculty development could be considered as an option to help improve the quality of narrative feedback in EPA observations.

## Author Contributions

**Rebecca Lee:** conceptualisation (lead), methodology (lead), formal analysis (equal), investigation (lead), resources (lead), writing – original draft (lead), writing – review and editing (equal), project administration (lead). **Neil Dhami:** formal analysis (equal), writing – review and editing (equal). **William Gibson:** formal analysis (equal), writing – review and editing (equal). **Deena M. Hamza:** supervision (supporting), writing – review and editing (equal), resources (equal). **Anna E. Oswald:** supervision (supporting), writing – review and editing (equal). **Mandy Moffat:** supervision (lead), writing – review and editing (equal).

## Funding

The authors have nothing to report.

## Ethics Statement

Ethical approval was obtained from the institutional Research Ethics Board and Trainee Research Access Committee (institutional requirement when requesting student data).

## Conflicts of Interest

The authors declare no conflicts of interest.

## Supporting information


**Appendix S1:** Association of Faculties of Medicine of Canada Core EPAs.

## Data Availability

The data that supports the findings of this study are available on request from the corresponding author (R.L.). The data are not publicly available due to containing information that could compromise the privacy of the research participants.
