# Supplementary material for: Assessing the Quality of Narrative Feedback in Entrustable Professional Activities Using the EFeCT Feedback Scoring Tool
Source: Clin Teach. 2025 Dec 8;23(1):e70295. doi: 10.1111/tct.70295 (PMC12685790; doi:10.1111/tct.70295)
Supplement: Supplementary file 1 — Appendix S1: Association of Faculties of Medicine of Canada Core EPAs. [file TCT-23-e70295-s001.docx]

**Appendices**

**Appendix A**

| **Association of Faculties of Medicine of Canada Core EPAs** | |
| --- | --- |
| **EPA** | **Description** |
| EPA 1 | Obtain a history and perform a physical examination adapted to the patient’s clinical situation |
| EPA 2 | Formulate and justify a prioritized differential diagnosis |
| EPA 3 | Formulate an initial plan of investigation based on the diagnostic hypotheses |
| EPA 4 | Interpret and communicate results of common diagnostic and screening tests |
| EPA 5 | Formulate, communicate and implement management plans |
| EPA 6 | Present oral and written reports that document a clinical encounter |
| EPA 7 | Provide and receive the handover in transitions of care |
| EPA 8 | Recognize a patient requiring urgent or emergent care, provide initial management and seek help |
| EPA 9 | Communicate in difficult situations |
| EPA 10 | Contribute to a culture of safety and improvement |
| EPA 11 | Perform general procedures of a physician |
| EPA 12 | Educate patients on disease management, health promotion and preventive medicine |

Association of Faculties of Medicine of Canada, 2019
